# Performance prediction of crosses in plant breeding through genotype by environment interactions

**DOI:** 10.1038/s41598-020-68343-1

**Published:** 2020-07-13

**Authors:** Javad Ansarifar, Faezeh Akhavizadegan, Lizhi Wang

**Affiliations:** Department of Industrial and Manufacturing Systems Engineering, Iowa State University, Ames, IA 50011 USA

**Keywords:** Machine learning, Plant breeding

## Abstract

Performance prediction of potential crosses plays a significant role in plant breeding, which aims to produce new crop varieties that have higher yields, require fewer resources, and are more adaptable to the changing environments. In the 2020 Syngenta crop challenge, Syngenta challenged participants to predict the yield performance of a list of potential breeding crosses of inbreds and testers based on their historical yield data in different environments. They released a dataset that contained the observed yields for 294,128 corn hybrids through the crossing of 593 unique inbreds and 496 unique testers across multiple environments between 2016 and 2018. To address this challenge, we designed a new predictive approach that integrates random forest and an optimization model for G $\times$ E interaction detection. Our computational experiment found that our approach achieved a relative root-mean-square-error (RMSE) of 0.0869 for the validation data, outperforming other state-of-the-art models such as factorization machine and extreme gradient boosting tree. Our model was also able to detect genotype by environment interactions that are potentially biologically insightful. This model won the first place in the 2020 Syngenta crop challenge in analytics.

## Introduction

Meeting the food demands of the world’s growing population is one of the most significant challenges that society is facing, especially due to the continuously changing climate^1^. Various approaches have been proposed to improve food production and security, including optimizing planting regime, sustainable farming practices, traits introgression, and modeling of plant physiology and ecology. In particular, optimizing the plant breeding process has been recognized as a promising area to improve global agrarian output with limited resources^2–4^. One of the most challenging decisions that plant breeders have to make is the selection of breeding parents for crosses^5^. For hybrid plant breeding, breeders make the best biparental crosses with high-yield potentials and test the hybrids’ yield performance by planting them in multiple locations and weathers. The empirical breeding process of predicting, planting, and evaluating biparental combinations is expensive, labor-intensive, and time-consuming, which is why scientists are turning to artificial crosses to help the breeders predict and select promising breeding parents for hybridization. The 2020 Syngenta crop challenge was a recent effort by the agriculture industry to address such a challenge with realistic datasets. The goal of this challenge is to predict the yield performance of inbred-tester combinations in a given test set.

Many classic models have been used for prediction and selection of parents for crosses, including, clustering technique^6^ as analysis of genetic diversity of hybrids, mixed models^5, 7, 8^, best linear unbiased prediction (BLUP)^9, 10^, ridge regression and the genomic best linear unbiased predictor (GBLUP)^11^, and regression methods such as ridge^12–14^ as predictor of cross performance of untested crosses, genetic relationship^15^ as assessment of yield performance of hybrid combinations.

More recently, machine learning models have been applied to predict yield performances of crosses. For example, González-Camacho et al.^16^ developed random forest, neural networks, and support vector machine (SVM) for predicting genomic performance. Montesinos-López et al.^17^ applied SVM, neural network, and BLUP in the genomic selection process. A probabilistic neural network was applied for genome-based prediction of corn and wheat in González-Camacho et al.^18^. Basnet et al.^19^ and Jiang et al.^20^ developed G $\times$ E interactions models for grain yield prediction using the genomic general combining ability (GCA) and specic combining ability (SCA) and their interactions with environments. Acosta-Pech et al.^21^ were the first to propose an extension of the models of Technow et al.^22^ and Massman et al.^23^ by combing the G $\times$ E model with the reaction norm model proposed by Jarquín et al.^24^. They used an interaction-based model with the interactions between SCA and GCA effects and environment for genomic predictions. State-of-the-art machine learning models have also been used for crop yield prediction, including stepwise multiple linear regression^25^, neural networks^25, 26^, convolutional neural networks^27, 28^, recurrent neural networks^28^, multiple regression^26^, random forest^29^, weighted histograms regression^30^, and association rule mining and decision tree^31^.

In this paper, we propose a new model for predicting the yield performance of new hybrids based on historical data of other hybrids. This model integrates a random forest with a combinatorial optimization-based interaction-detection model and attempts to combine their strengths. The random forest model^32^ is known for its capability to approximate general form nonlinear relationships among the variables. On the other hand, the interaction-detection model originated from a recently published algorithm^33^ that has been shown to be particularly effective in detecting epistatic type of interactions. Our model extends that algorithm to the detection of genotype by environment interactions (G $\times$ E).

Our computational results using the 2020 Syngenta crop challenge data suggested that the proposed model can accurately predict the performance of untested cross combinations of inbreds and testers. Moreover, results of our prediction model can also reveal biologically meaningful insights, such as the best hybrids for specific environments.

## Problem definition

Most of the effort in a breeding program is related to evaluating inbreds by crossing to another inbred known as a tester. According to the problem statement of the 2020 Syngenta crop challenge, “it is a plant breeder’s job to identify the best parent combinations by creating experimental hybrids and assessing the hybrids’ performance by ‘testing’ it in multiple environments to identify the hybrids that perform best.” While the yield performance of a hybrid is largely related to the parents, it is also affected by many factors that are hard to predict, such as heterosis and interactions between genotype and the environment.

The objective of the 2020 Syngenta crop challenge was to design a model for predicting the yield performance of a list of inbred-tester combinations based on historical datasets that included yield, genetic group, and pedigree information of hybrids collected in different environments over a number of years. If successful, this challenge will stimulate novel design of predictive models and algorithms for yield prediction of inbred-tester combinations and progeny testing of inbreds, which will help breeders make the most promising crosses without having to rely on large-scale trial-and-error that is expensive, labor intensive, and time consuming. The 2020 Syngenta crop challenge released the following dataset for commercial corn.

### Training dataset

- Yield: Historical yield performances were measured for 10,919 unique biparental hybrids. To provide realistic data without revealing proprietary information, actual yield values were anonymized to make the average and standard deviation of yields approximately 1.0 and 0.1, respectively. The range of the yields was from 0.0472 to 1.8001.
- Genetic clusters: No genetic marker information was available, but the genetic clusters of 593 unique inbreds and 496 unique testers were provided. Syngenta grouped the inbreds and testers into some clusters according to their genetic similarities using internal methods. There were 14 inbred clusters and 13 tester clusters.
- Environment: Out of a total of 593 $\times$ 496 = 294,128 possible combinations of inbred-tester crosses, the training data included 10,919 unique hybrids that were planted across 280 locations between 2016 and 2018, each year with a unique set of weather conditions. The information that we had for the environment is 280 location IDs and 3 years such that there were 599 unique location-weather combinations in the training set. The total number of unique hybrids-location-weather combinations was 155,765, some of which had multiple replications, so the total number of yield records was 199,476. However, this training dataset represents only 0.08% of all possible 593 $\times$ 496 $\times$ 280 $\times$ 3 = 247,067,520 hybrids-location-weather combinations.

### Test dataset

The test dataset includes a set of inbred-tester combinations whose yield performances need to be predicted. The environments in which these hybrids would be grown were not specified in the crop challenge.

### Evaluation criteria

The evaluation criteria for the 2020 Syngenta crop challenge in analytics were “accuracy of the predicted values in the test set based on root mean squared error, simplicity and intuitiveness of the solution, clarity in the explanation, and the quality and clarity of the finalist’s presentation at the 2020 INFORMS Conference on Business Analytics and Operations Research.” Our model won the first place in this competition. For this paper, we evaluated the proposed model in terms of prediction accuracy. Because we did not have access to the ground truth yield of the test dataset, we divided the given dataset to training and validation subsets using tenfold cross-validation (CV). Then, we used the average performance of the proposed model as the evaluation criteria.

## Method

### Data preprocessing

We defined the input variable *X* as one-hot coding of hybrid-location-weather combinations and the output variable *y* as the corresponding yield. To accommodate this definition, four types of training data were converted to binary using the one-hot coding preprocessing: inbred and tester indices, genetic cluster, location ID, and weather. For those hybrid-location-weather combinations with multiple replications, the average yield was used as the output data. As such, the training data has a dimension of 155,765 observations by 1,399 (593 inbreds $+$ 496 testers $+$ 14 inbred clusters $+$ 13 tester clusters $+$ 280 locations $+$ 3 years of weather) one-hot coding variables.

### Proposed model and algorithm

**Figure 1:**
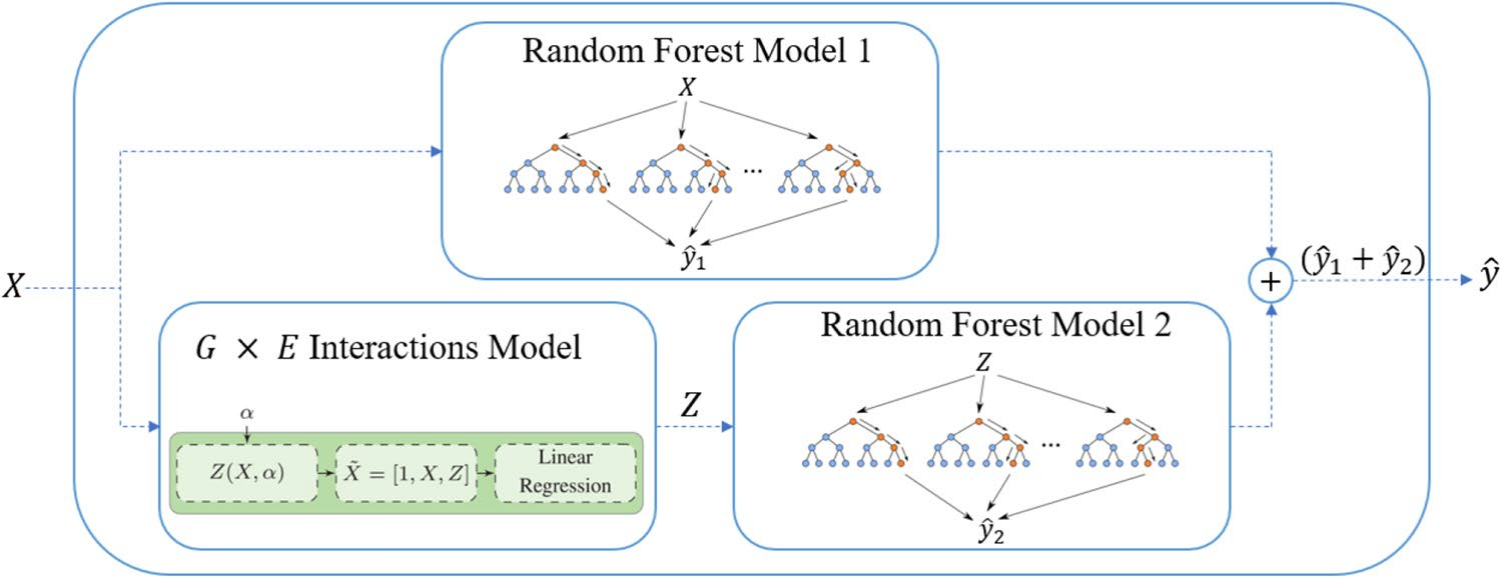
The test process of proposed model. This plot was created with Microsoft PowerPoint (Version 16.0.12827.20200 32-bit).

We proposed a hybrid model for this challenge, which combines random forest with G $\times$ E interaction detection techniques. The overview of the model is diagramed in Fig. 1. This model consists of three main components: a random forest model that captures the complex nonlinear relationship between input and output variables, a G $\times$ E interaction detection model that captures interactions among hybrid, location, and weather variables, and another random forest model that utilizes the interactions to augment the prediction performance of the first random forest model. Details of these components are described in the rest of this section.

#### Random forest model 1

Random forest^32^ is an ensemble learning model that can be used for classification or regression by constructing a multitude of decision trees. To grow each tree, a random subset of features is selected along with replacement sampling (bootstrap sampling) used to select different subsets of the observations. Therefore, observations in the dataset that were not included in the bootstrapped samples are considered as out-of-bag observations, and the performance of the tree is evaluated by the average out-of-bag error. Due to the builtin component of cross-validation, the random forest is less prone to overfitting.

The random forest model 1 takes the one-hot matrix *X* as input and predicts the corresponding yield performance ${\hat{y}}$ as output. This model is sensitive to three hyperparameters: the number of trees should be large enough to stabilize the error rate and small enough to be tractable; the number of features controls tree correlation, and the node size (minimum size of terminal nodes) determines the complexity of the individual trees. A tenfold CV was used to partition dataset to training and validation subsets. For each fold, we used the training subset for training and parameter tuning. A fivefold CV over train partition for each fold was applied to tune the parameters. Table 1 gives the values of these hyperparameters using a fivefold CV over the whole dataset to get the best values that lead to good performance on the validation dataset.

**Table 1:** Tuned hyperparameters for the random forest model 1.

Hyperparameters	Value
Number of trees	1000
Number of features	100
Node size	10

#### G $\times$ E interactions model

The random forest model has the capability to approximate nonlinear relationships among the variables. It grows many classification trees by randomly selecting subsets of features. As such, this model is ineffective in discovering specific combinations of features that have the most significant interactions. Therefore, we also introduced a combinatorial optimization-based model to augment the random forest by strategically searching for G $\times$ E interactions.

The G $\times$ E interactions model was designed to detect interactions among specific hybrid, location, and weather variables. This model is built off of a recently published algorithm^33^, which was designed to detect genetic interactions in the form of epistases. The algorithm was found to be effective in detecting multiple interactions involving multiple variables. The G $\times$ E interactions model considers yield as a linear function of input variables and their interactions, shown as follows.

1 $$\begin{aligned} {\hat{y}}_i =\beta _0 + \sum _{j=1}^p X_{i,j} \beta _j + \sum _{k=1}^K b_k Z_{i,k} + \epsilon _i. \quad \forall i \in \{1,...,n\} \end{aligned}$$

Here,

- $X_{i,j} \in \{0, 1\}$ is the one-hot input variable *j* of observation *i*,
- ${\hat{y}}_i$ is the yield of observation *i*,
- $Z_{i,k} \in \{0, 1\}$ indicates whether or not observation *i* receives interaction *k*,
- $\beta _0$, $\beta _j$, and $b_k$ are the effects of baseline, variable *j*, and interaction *k*, respectively, and
- $\epsilon _i$ is random noise for observation *i*.

In this model, the interactions are defined by a matrix $\alpha$, which has a dimension of $K \times p$, where *K* is the number of interactions that the proposed model tries to decipher and *p* is the number of variables. Each column of this matrix corresponds to a variable and each row corresponds to an interaction. Moreover, each element of matrix $\alpha$ can take three possible values 0, 0.5, 1. If $\alpha _{k,j}=0$, then interaction *k* requires that variable *j* be 0 ($X_{i,j} = 0$) for any individual *i* to receive this effect. If $\alpha _{k,j}=1$, then interaction *k* requires that variable *j* be 1 ($X_{i,j} = 1$) for any individual *i* to receive this effect. If $\alpha _{k,j}=0.5$, then variable *j* is not involved in interaction *k*. Given matrix $\alpha$, the matrix *Z* can be subsequently calculated to determine whether or not the individuals receive the interactions. The dimension of the binary matrix *Z* is $n \times K$, with each row corresponding to one individual and each column corresponding to one interaction. If $Z_{i,k}=1$, then individual *i* receives the interaction *k*, and $Z_{i,k}=0$ otherwise. This complex relationship can be captured mathematically as: individual *i* receives interaction *k* $(Z_{i,k}=1)$ if and only if $X_{i,j} + \alpha _{k,j} \ne 1$, or equivalently $X_{i,j}=\alpha _{k,j}$, for each variable *j*.

The key to model (1) is to find *Z* from a given training dataset $(X^\text {Train}, y^\text {Train})$, which requires the estimation of the number of interactions and the combination of variables that are involved in each interaction. When *Z* has been determined, model (1) reduces to a multiple linear regression that is easy to solve and interpret.

**Figure 2:**
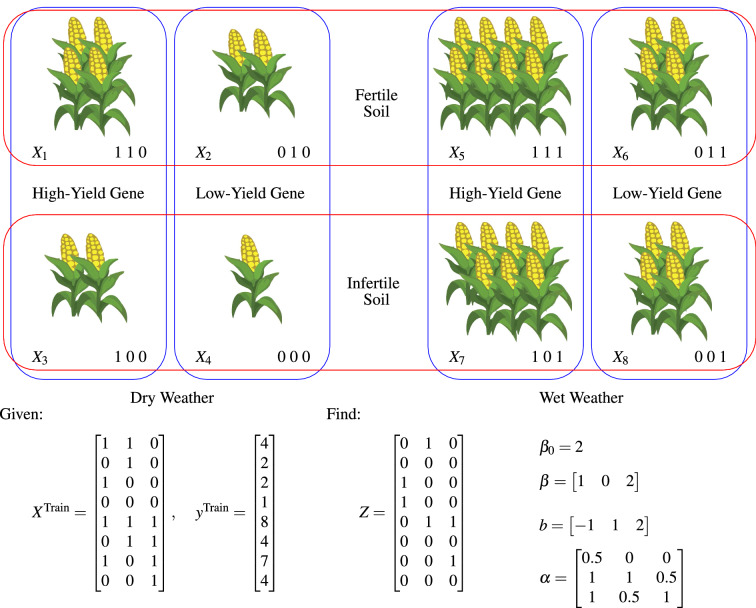
An illustrative example of G $\times$ E interactions. This plot was created with Microsoft PowerPoint (Version 16.0.12827.20200 32-bit) and MiKTeX (Version 2.9.7206).

Figure 2 illustrates an over-simplified example of G $\times$ E interactions on corn yield. The given training data gives the yield of $n = 8$ corn plants with all possible combinations of $p = 3$ variables: high-yield (1) or low-yield (0) gene, fertile (1) or infertile (0) soil, wet (1) or dry (0) weather. No random noise was added to simplify the illustration. The figure shows the solution to the model (Eq. 1). Matrix *Z* has three columns, indicating three interactions.

- The first interaction is triggered by infertile soil $(\alpha _{1,2}=0)$ and dry weather $(\alpha _{1,3}=0)$, reducing yield by 1 ($b_1 = -1$). Plants #3 and #4 receive this effect, indicated by the first column of matrix *Z*.
- The second interaction is triggered by high yield gene $(\alpha _{2,1}=1)$ and fertile soil $(\alpha _{2,2}=1)$, increasing yield by 1 ($b_2 = 1$). Plants #1 and #5 receive this effect, indicated by the second column of matrix *Z*.
- The third interaction is triggered by high yield gene $(\alpha _{3,1}=1)$ and wet weather $(\alpha _{3,3}=1)$, increasing yield by 2 ($b_3 = 2$). Plants #5 and #7 receive this effect, indicated by the third column of matrix *Z*.

The rest of the solution indicates that the baseline yield is $\beta _0=2$, the high yield gene, and wet weather contribute additional $\beta _1=1$ and $\beta _3=2$, respectively, and the fertile soil has no additive effect ($\beta _2=0$).

In our model, a similar approach is used to detect interactions among hybrid, soil, and weather at a much larger scale with $n = 155,765$ and $p = 1,399$. To overcome the computational challenges, we used a similar heuristic algorithm as in^33^, which had three desirable features: (1) it used cross-validation to avoid-overfitting; (2) it was able to find local optimal solutions efficiently; and (3) it could be parameterized to balance computation time and solution quality.

#### Random forest model 2

Although the interaction model can decipher the interactions between binary predictors, it cannot find more complex nonlinear function of interactions. Hence, we feed the results of the G $\times$ E model into another random forest to identify more complex nonlinear interactions. Random forest model 2 was designed to predict the residual prediction from random forest model 1. Let ${\hat{y}}_1$ and ${\hat{y}}_2$ denote the predictions from random forest models 1 and 2. The overall model output ${\hat{y}}_1 + {\hat{y}}_2$ will provide a more accurate prediction of yield, *y*, than ${\hat{y}}_1$ if ${\hat{y}}_2$ can be trained to estimate $y - {\hat{y}}_1$.

To achieve this objective, we feed matrix *Z* from the G $\times$ E interactions model to random forest 2 to predict not only linear G $\times$ E interactions described in matrix *Z* but also more complex and nonlinear interactions. This model is trained using the residual of $y - {\hat{y}}_1$ to improve its accuracy. The tuned hyperparameters for the random forest model 2 are reported in Table 2. The same process as the random forest model 1 was applied to tune hyperparameters.

**Table 2:** Tuned hyperparameters for the random forest model 2.

Hyperparameters	Value
Number of trees	1000
Number of features	20
Node size	10

The proposed model combines the strengths of combinatorial optimization in identifying G $\times$ E interactions and random forest in producing accurate predictions using complex and nonlinear functions. As such, it is a trade-off between insight and accuracy. It will be shown in the computational experiments that this hybrid model produced more insightful and accurate predictions than using either model alone.

## Quantitative results

In this section, we report the results of our computational experiments, which were designed to test the performance of the proposed algorithm with respect to other benchmark approaches.

### Prediction accuracy

To show the performance of the proposed model, it was compared with models from the literature, which are summarized as follows:

- A multiple linear regression model was trained using the glmnet^34^ package in R statistical software (version 3.4.4).
- The multi-way interacting regression via factorization machines (MiFM)^35^ was implemented in Python by the authors.
- An extreme gradient boosting tree (XGBoost)^36^ model was trained using the xgboost^36^ package in R, which was an efficient and scalable implementation of gradient boosting framework. Three hyperparameters were tuned using fivefold cross validation (without data leakage): “nrounds”, “eta”, and “gamma”.
- A G $\times$ E interactions model^33^ was implemented in MATLAB (Version 2018a), which used heuristic algorithms to detect multi-way and multi-effect epistasis (interactions between binary variables). It is equivalent to the G $\times$ E interactions model without integrating with the random forest models.
- A random forest^32^ was trained using the ranger^37^ packages in R, which was an ensemble of decision trees and trains with the bagging method, equivalent to the random forest model 1 without the interaction model and the random forest model 2 in our proposed model. Three hyperparameters were tuned using fivefold cross-validation: the number of trees, number of features, and node size.
- The proposed model was implemented in MATLAB (Version 2018a).

Three metrics were used for evaluating and comparing the predictive models’ performances: RMSE, which presents the difference between predicted and observed values, Mean Absolute Error (MAE), which measures the average magnitude of the prediction errors, without considering their direction, and $R^2$, the coefficient of determination defined as the proportion of the variance in the response variable that is explained by independent variables. Because the ground truth of the test dataset was never released, we partitioned the training dataset into training and validation subsets in a tenfold CV manner. For each fold, we tuned the parameters and trained the models using the training set, and then their performances were evaluated using the validation set. We made sure that no validation data was leaked in the model training process. The average RMSE, MAE, and $R^2$ values over ten partitions for the six algorithms are reported in Table 3. These results indicate that the proposed model outperformed other algorithms in all measures. Since the random forest model was part of our proposed model and it outperformed the first four machine learning algorithms, these results indicated the effectiveness of both the random forest method and our G $\times$ E interactions detection model.

**Table 3:** Average RMSE, MAE, and $R^2$ of six algorithms for yield prediction. A 10-fold cross-validation on the training dataset was used for algorithm performance evaluation, since the ground truth yield of the test dataset was never released.

Model	Train			Validation		
	RMSE	MAE	${R}^2$	RMSE	MAE	${R}^2$
Linear regression	0.1016	0.1009	0.1047	0.1026	0.0851	0.0866
Factorization machine	0.0740	0.0676	0.4855	0.0984	0.0765	0.1578
Xgboost	0.0790	0.0735	0.4581	0.0996	0.0806	0.1388
G $\times$ E	0.0740	0.0706	0.4902	0.0980	0.0744	0.1623
Random forest	0.0737	0.0673	0.5283	0.0976	0.0723	0.1738
Proposed model	0.0548	0.0523	0.7386	0.0869	0.0648	0.3448

**Figure 3:**
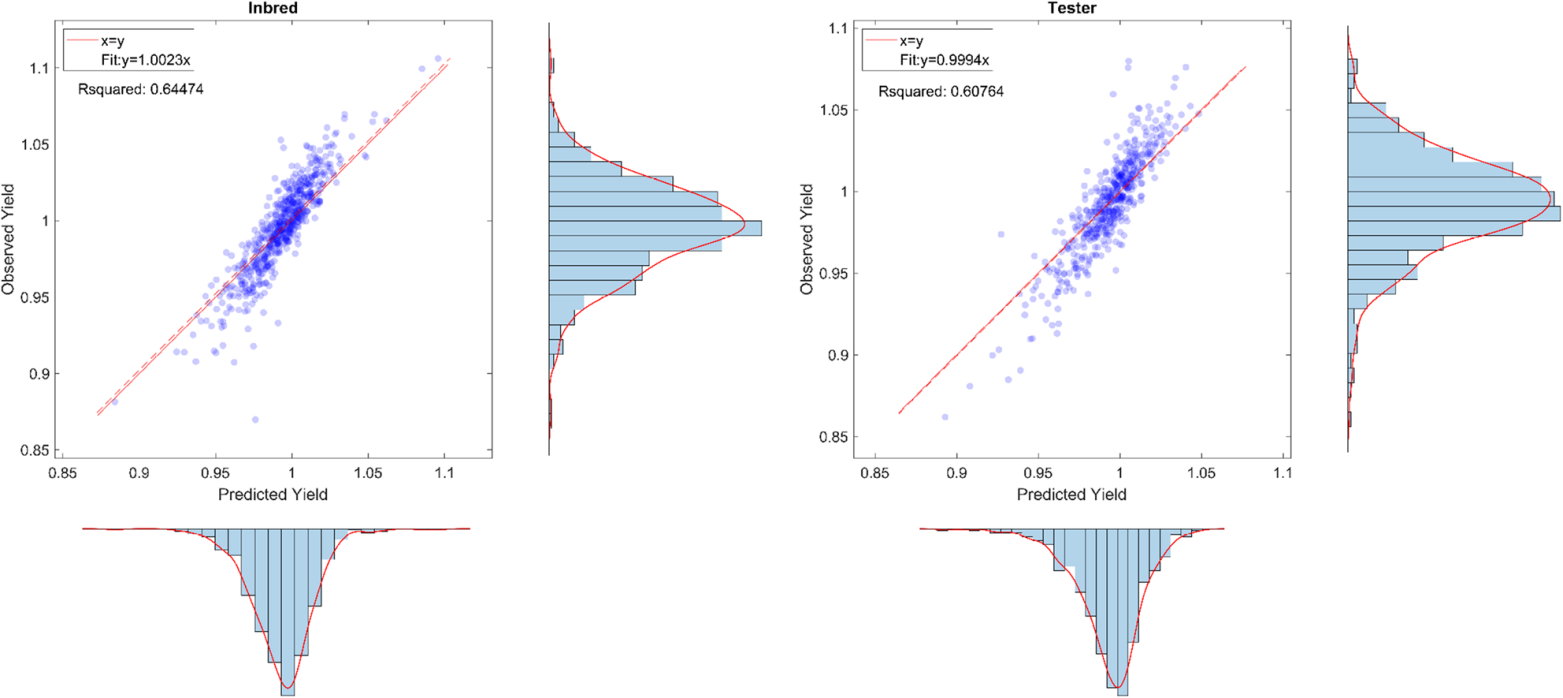
The left and right plots indicate the plots of the average observed yield versus the average predicted yield for performances of inbreds and testers, respectively. These plots were created with MATLAB R2018a (Version 9.4.0.813654 64-bit).

The performance of the proposed model is also illustrated in Fig. 3, which plots the average predicted yields against actual observations for all inbreds and testers. The results suggest that our proposed model’s prediction is close to the observation, both on average and in terms of probability density distributions.

We also examined the consistency of top and bottom inbreds and testers selected based on our prediction model against those based on observations. Out of the top 29 (5%) inbreds among all 593 inbreds with the highest average yield selected by our model, 21 of them were consistent with those selected based on actual observations. Similarly, out of the top 24 (5%) testers among all 496 testers with the highest average yield selected by our model, 17 of them were consistent with those selected based on actual observations. The counterpart consistency ratios for the bottom 5% inbreds and bottom 5% testers are $\dfrac{22}{29}$ and $\dfrac{16}{24}$, respectively. The predicted and observed average yield for the 14 inbred clusters and 13 tester clusters are summarized in Table 4.

**Table 4:** Predicted and observed average yield of 14 inbred clusters and 13 tester clusters.

Average yield	Inbred cluster													
	1	2	3	4	5	6	7	8	9	10	11	12	13	14
Predicted	1.010	1.010	1.011	0.997	1.007	0.991	1.002	0.993	0.997	0.990	0.986	0.991	0.992	0.998
Observed	1.006	1.020	1.007	0.992	1.003	0.981	0.999	0.988	0.990	0.991	0.984	0.992	0.996	0.996

Average yield	Tester cluster													
	1	2	3	4	5	6	7	8	9	10	11	12	13	
Predicted	0.999	1.002	0.999	0.992	1.004	0.993	1.005	1.005	0.998	0.981	0.999	0.992	1.001	
Observed	0.995	0.996	0.994	0.992	1.003	0.997	1.001	1.001	0.998	0.980	1.005	0.975	0.996	

### Genotype and environment interactions

**Figure 4:**
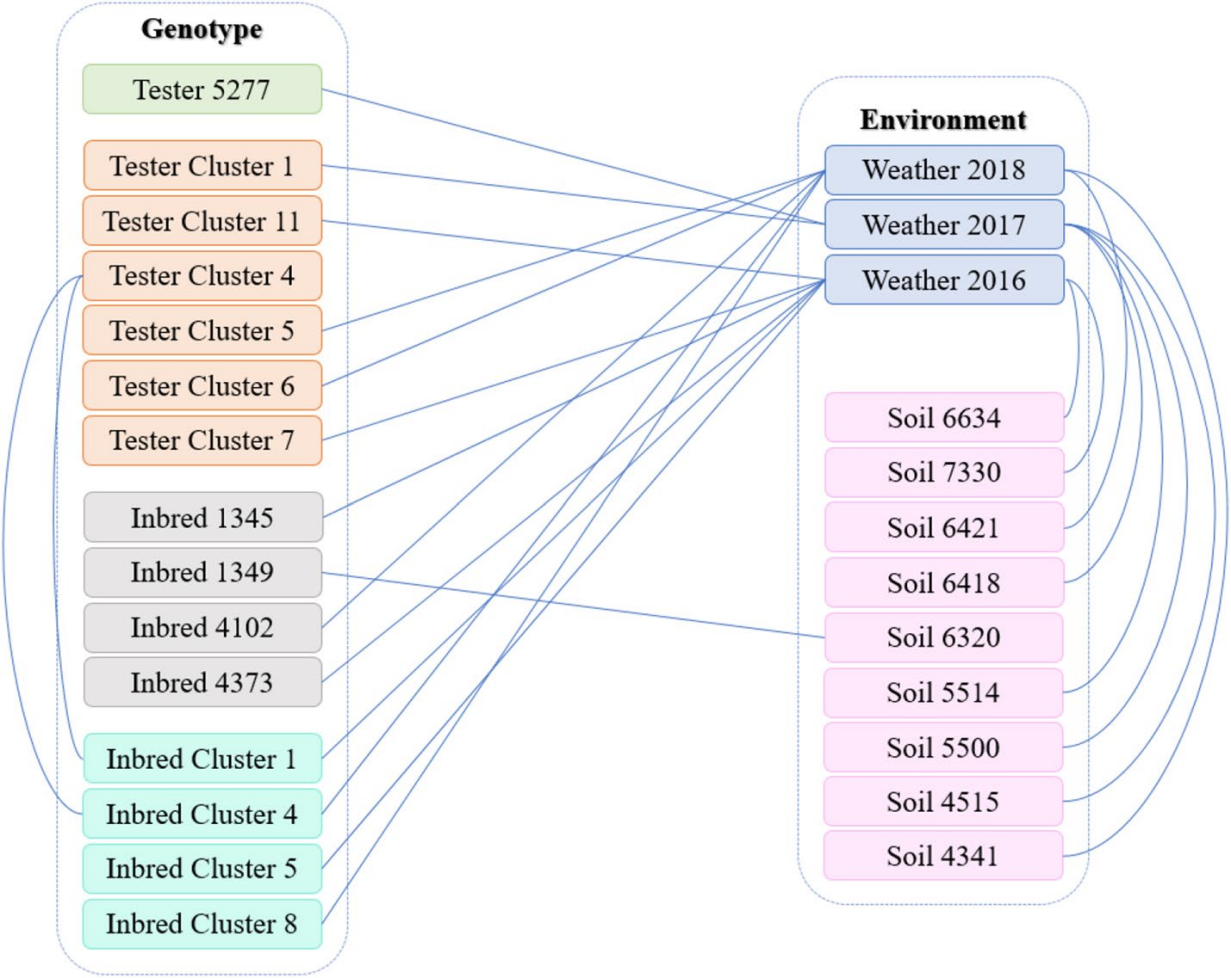
Two-way interactions. Each line shows the two-way interaction between two variables. This plot was created with Microsoft PowerPoint (Version 16.0.12827.20200 32-bit).

**Figure 5:**
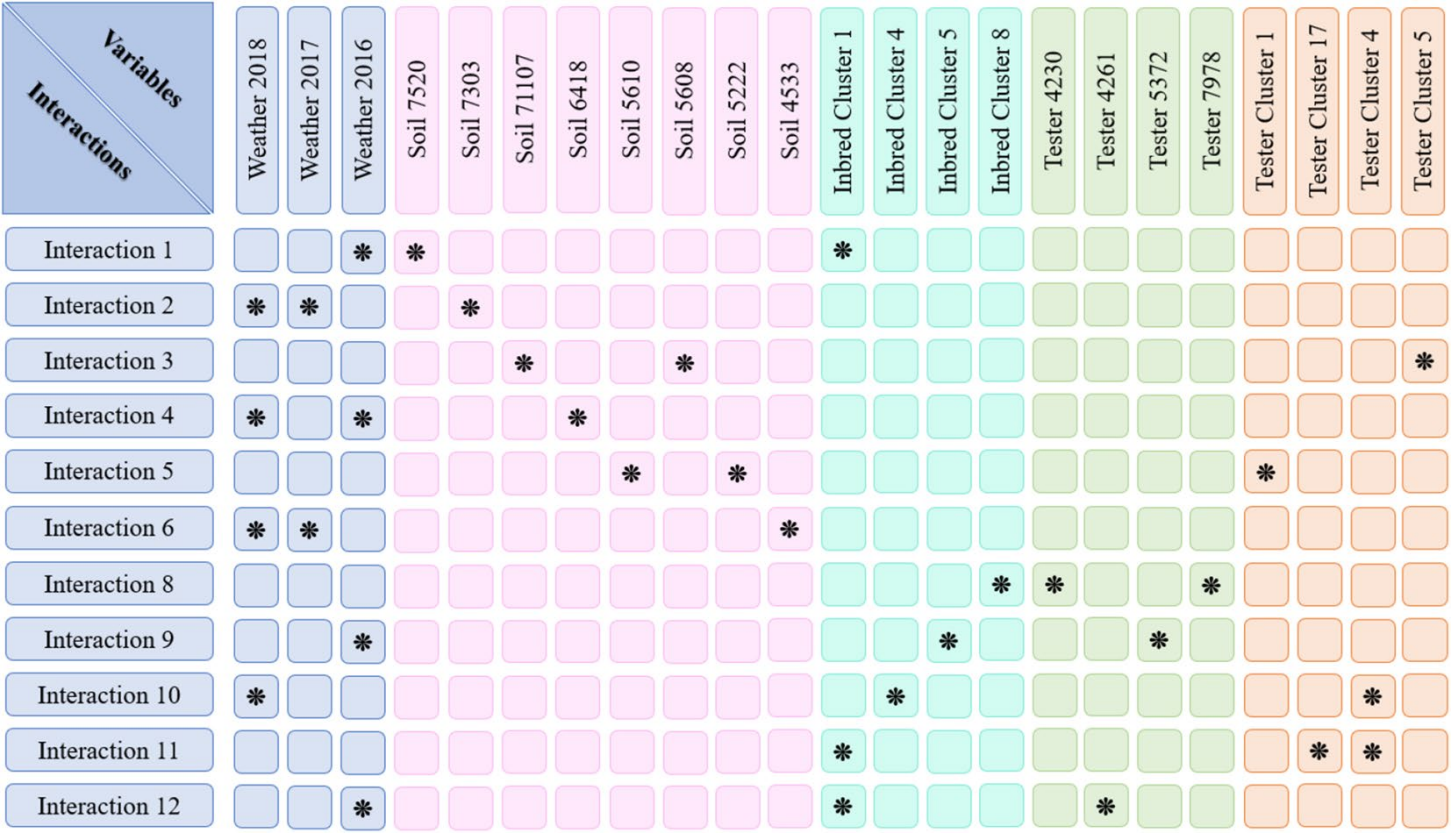
Three-way interactions. Each row indicates the three-way interaction between three variables. The star markers in each row indicate which variables involve in the interaction. This plot was created with Microsoft PowerPoint (Version 16.0.12827.20200 32-bit).

The proposed model was able to provide not only accurate yield prediction but also genotype and environment interactions that could be biologically insightful. Figures 4 and 5 show the two-way and three-way interactions between variables, respectively. The results indicate that weather variables involve in more interactions following soil and genotype.

### Optimal biparental crosses

**Figure 6:**
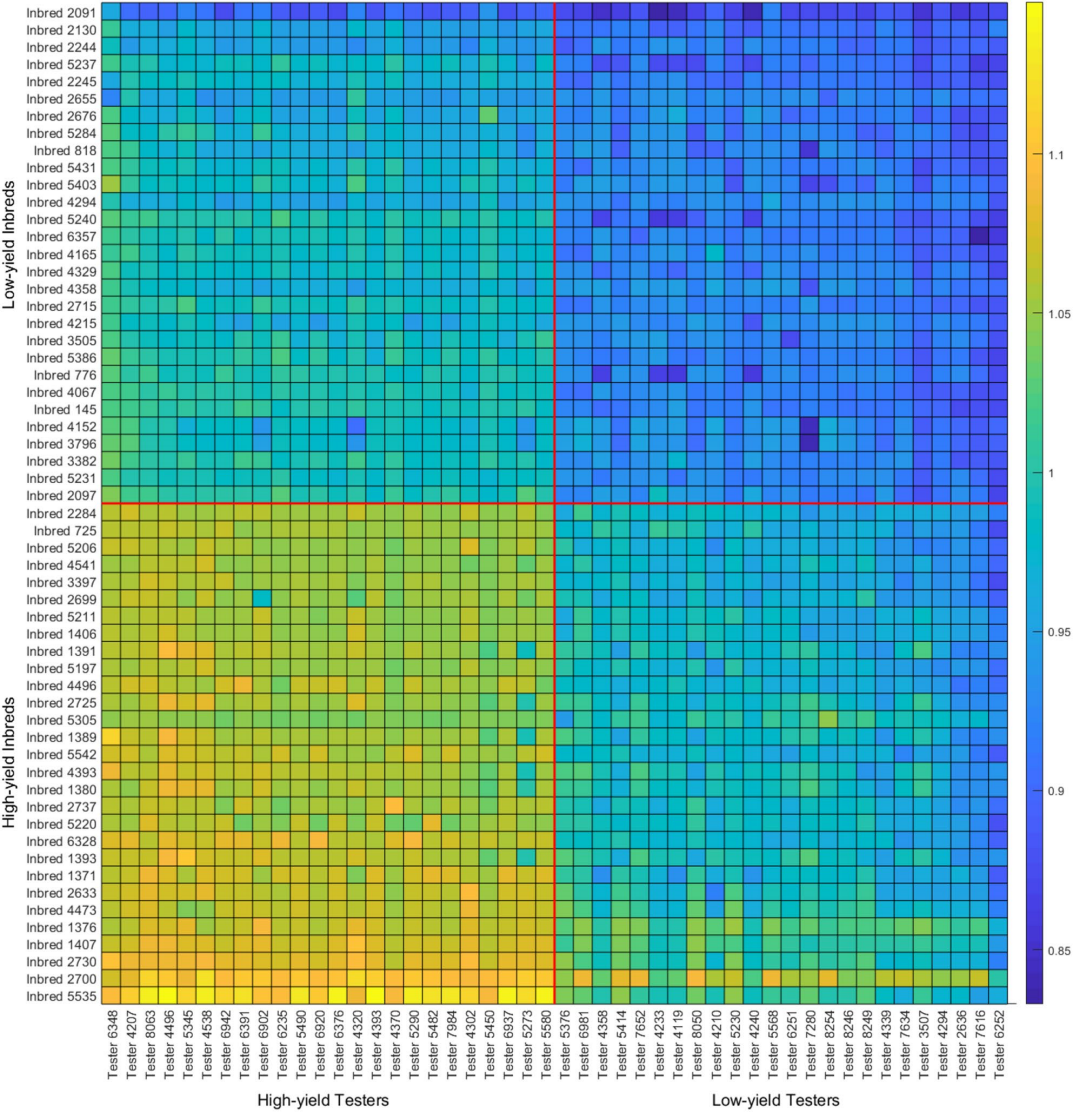
Predicted yield performances for combinations of the top and bottom 5% of inbreds and testers. This plot was created with MATLAB R2018a (Version 9.4.0.813654 64-bit).

To shed light on optimal biparental crosses between the given inbreds and testers, we used the proposed model to predict the yield performance of all combinations of testers and inbreds in different years and locations. Then, we ranked them based on average yield performance over all years and locations. The results of the top and bottom 5% of inbred-tester combinations (combinations of top and bottom 29 inbreds with top and bottom 24 testers) are illustrated in Figure 6, which can help breeders predict the most promising crosses. The average yields for four combinations of crosses are given in Table 5. These results appear to suggest that testers have a slightly higher weight in determining the yield performance of their progeny.

**Table 5:** Average yield performance of combinations of high- and low-yield testers and inbreds.

	High-yield tester	Low-yield tester
Low-yield inbred	1.0098	0.9457
High-yield inbred	1.0625	0.9789

## Conclusion

We proposed a new model to address the 2020 Syngenta crop challenge, which combines random forest with an G $\times$ E interactions model to predict yield performance of inbreds and testers based on historical yield data in multiple years and environments. Random forest model has been found to be an effective and powerful machine learning model for prediction, yet it has its limitations in the degrees and types of interactions among the predictors. Based on a recently published algorithm for detecting multi-way and multi-effect epistatic effects, the G $\times$ E interactions model captures both linear and nonlinear interactions of the genotype by environment effects. The combination of random forest and the G $\times$ E interactions model was found to be effective in predicting yield performances of inbred-tester combinations in our computational study using tenfold validation, achieving a 0.0869 validation RMSE, 0.0648 validation MAE, and 0.3448 R-squared value, outperforming four other popular machine learning algorithms as the benchmark. Moreover, our proposed model was also more explainable than other machine learning models by yielding genotype by environment interactions. Results from our proposed model will be able to help breeders test progeny and identify the best parent combinations to produce new hybrids with improved yield performances.

## Data Availability

The data analyzed in this study was provided by Syngenta for 2020 Syngenta crop challenge. We accessed the data through annual Syngenta crop challenge. During the challenge, September 2019 to January 2020, the data was open to the public. Researchers who wish to access the data may do so by contacting Syngenta directly (https://www.ideaconnection.com/syngenta-crop-challenge/challenge.php).
